# Placental abnormalities of malperfusion, inflammation, and meconium are associated with neonatal pulmonary vascular disease

**DOI:** 10.1016/j.placenta.2026.06.024

**Published:** 2026-07-10

**Authors:** Stephanie M. Tsoi, Cynthia Gasper, Emin Maltepe, Megan A. Chidboy, Nida Ozarslan, Christine A. Blauvelt, Sirirak Buarpung, Shannon Cheung, Martina Steurer, Roberta L. Keller, Jeffrey R. Fineman, Stephanie L. Gaw

**Affiliations:** a Division of Critical Care Medicine, Department of Pediatrics, University of California San Francisco, San Francisco, CA, USA; b Department of Pathology and Laboratory Medicine, University of California, San Francisco, CA, USA; c Division of Neonatology, Department of Pediatrics, University of California San Francisco, San Francisco, CA, USA; d Division of Maternal-Fetal Medicine, Department of Obstetrics, Gynecology & Reproductive Sciences, University of California, San Francisco, CA, USA; e Department of Epidemiology and Biostatistics, University of California San Francisco, San Francisco, CA, USA; f Cardiovascular Research Institute, University of California San Francisco, San Francisco, CA, USA

**Keywords:** Persistent pulmonary hypertension of the newborn, Pulmonary vascular development, Placenta, Maternal-fetal environment

## Abstract

**Objectives:**

The primary objective was to elucidate whether placental histopathology representative of intrauterine insults during pulmonary vascular development are associated with a diagnosis of persistent pulmonary hypertension of the newborn (PPHN). The secondary objective was to compare placental histopathologic lesions across PPHN etiologies.

**Study design:**

We conducted a case-control study of mother–infant dyads ≥35 weeks gestation who delivered at a tertiary care center between 2020 and 2025. Cases were infants diagnosed with PPHN and treated with inhaled nitric oxide; unaffected controls were infants without congenital anomalies. Placentas underwent blinded histopathologic review using standardized criteria. Multivariate logistic regression modeling was used to control for confounding maternal and infant factors.

**Results:**

106 placentas were analyzed (53 PPHN, 53 controls). Placental lesions were significantly more common in PPHN placentas, including fetal vascular malperfusion (30.2% vs 9.4%, p < 0.01), placental inflammation (66.0% vs 37.7%, p < 0.01), chronic presence of meconium (43.4% vs 15.2%, p < 0.01), and chorangiosis (7.6% vs 0%, p = 0.04). In adjusted analyses compared to controls, among 41 placentas with fetal development etiologies (e.g. congenital anomalies) of PPHN, fetal vascular malperfusion, placental inflammation and fetal inflammatory response were more common. Among 12 placentas with typical causes of PPHN (e.g. meconium aspiration syndrome), placental inflammation, maternal and fetal inflammatory responses, and meconium were more common.

**Conclusions:**

PPHN placentas demonstrate lesions of malperfusion, inflammation, and chronic meconium, suggesting a complex interplay between intrauterine hypoxia and inflammation as a potential mechanism for the abnormal pulmonary vascular development and function seen in PPHN.

## 1. Introduction

Persistent pulmonary hypertension of the newborn (PPHN) is due to the failure of the pulmonary vascular resistance to fall during the perinatal transition. It presents as hypoxic respiratory failure in infants within the first hours or days of life. The incidence of PPHN in late preterm and term infants is 0.18% of live births, with a 1-year mortality rate of 7.6% [1]. Multiple etiologic mechanisms may lead to PPHN, including the more common, typical perinatal causes (e.g. sepsis and meconium aspiration syndrome) and the less common fetal developmental causes (e.g. neonatal interstitial lung disease and lung hypoplasia syndromes such as congenital diaphragmatic hernia) [2].

The underlying mechanisms of PPHN are poorly understood. While PPHN from fetal developmental causes is often secondary to underdevelopment of the lung and its vasculature [3], typical PPHN is less well defined. Typical PPHN occurs in lungs that are thought to have developed normally throughout gestation, suggesting a late maladaptation of pulmonary vasculature. Knowledge from animal and human models of pulmonary arterial hypertension suggest that hypoxia, shear stress, inflammation, and genetic influences may contribute to abnormal pulmonary vascular structure and reactivity, depending on severity and duration of the adverse stimuli. Several studies have suggested that alterations in the fetal milieu and the placenta are associated with abnormalities in the pulmonary vasculature. Thureen and colleagues identified pulmonary vascular remodeling with arterial medial muscular hypertrophy in fatal meconium aspiration syndrome, accompanied by placental vascular malperfusion lesions [4]. Mestan and colleagues showed an association between placental maternal vascular underperfusion and development of late pulmonary hypertension complicating bronchopulmonary dysplasia (BPD) in a cohort of preterm newborns [5]. Finally, Fleck et al. demonstrated elevated inflammatory markers in cord blood of newborns with congenital diaphragmatic hernia (CDH) who had persistence of pulmonary hypertension at 2 weeks of age [6]. Thus, known mechanisms of pulmonary hypertension may be relevant in PPHN when active prenatally [7,8].

We hypothesize that the intrauterine environment has a crucial role in pulmonary vascular development, function, and transition. As the placenta is the most important organ during pregnancy, responsible for *in utero* gas and nutrient exchange as the interface between mother and fetus, it can reflect alterations in the intrauterine environment that we hypothesize may be associated with PPHN. Thus, the objective of this study was to investigate the placental abnormalities that precede PPHN to elucidate potential mechanisms of disease.

## 2. Materials and methods

This is a case-control study of mother-infant dyads who delivered between January, 2020 to May, 2025 at the University of California, San Francisco Birth Center. The Birth Center has a delivery service that is both community-based and, more often, referral-based, as it has a fetal diagnostic and interventional center with coordinated delivery and neonatal care for fetuses with anomalies and other conditions requiring specialized perinatal care. Cases were identified if an infant met all of the following criteria: had hypoxemic respiratory failure, diagnosed with PPHN, received inhaled nitric oxide treatment for at least 4 h, and had a placenta sent to pathology at the time of birth. Controls were infants without major congenital anomalies, delivered at the UCSF Birth Center during the same study period as the cases, and randomly selected from mothers with similar risk factors and who had consented to placenta donation. Available control placentas were matched for gestational age, infant sex, maternal gravidity, and method of delivery. The inclusion criteria for both cases and controls was gestational age greater than or equal to 35 weeks. This study was approved by the University of California, San Francisco institutional review board: PPHN cases were approved for a waiver of consent, given the retrospective study design. Controls provided written informed consent for sample collection and review of medical records (IRB # 21–33576, #20–32077).

### 2.1. Demographic and clinical data

Electronic medical records from mother-infant dyads were reviewed to obtain information on demographics, maternal co-morbidities, pregnancy complications, delivery information, and neonatal outcomes. Infant race was self-reported by the primary caregiver. Fetal growth restriction (FGR) was defined as having an abdominal circumference or estimated fetal weight below the tenth percentile for gestational age. Small for gestational age (SGA) was defined as having a birth weight below the tenth percentile for gestational age. Advanced maternal age (AMA) was defined as having a maternal age greater than or equal to 35 years. Cases of PPHN were further classified into two groups based on their etiology of disease: 1) typical PPHN (from perinatal causes such as meconium aspiration syndrome or sepsis, and idiopathic cases) and 2) developmental PPHN (from fetal developmental causes such as interstitial lung disease, pulmonary hypoplasia, or genetic syndromes) [2].

### 2.2. Placental collection and processing

At the time of delivery, placentas were sent for gross and microscopic histopathologic examination or collected for research and fixed in 10% buffered formalin. Two to four full-thickness sections from the chorionic plate to the basal plate were taken of each placenta. Sections underwent routine processing, were paraffin embedded, sectioned at 4 μm, and stained with hematoxylin and eosin. A single subspecialist pathologist (C.G.), who was blinded to clinical data except gestational age, reviewed the entirety of each slide under light microscopy (magnification 40 to 600x), and categorized the pathologic lesions according to the Amsterdam Criteria [9]. Placental pathologic findings of interest included any evidence of maternal vascular malperfusion, fetal vascular malperfusion, and inflammation (both maternal and fetal inflammatory response), as well as findings of meconium, chorangiosis, or intervillous thrombi (Table 1).

### 2.3. Statistical analysis

Descriptive data were reported as means with standard deviations or medians with IQR, and counts with percentages. Patients were included even if data were missing for some covariates. Characteristics of the PPHN groups were compared to each other (typical versus developmental) and PPHN cases to controls via *t*-test for continuous variables and by chi-squared analysis for categorical variables. The associations between PPHN and placental findings were evaluated by univariate logistic regression modeling. When comparing all PPHN placentas to controls, multivariate logistic regression modeling was used to control for potential confounding maternal (gestational hypertension, AMA, maternal obesity) and fetal factors (FGR). Secondary exploratory stratified analyses comparing placental findings for each etiology of PPHN (developmental or typical) to controls included chi-squared analysis and logistic regression modeling. Multivariate logistic regression modeling for developmental PPHN included the three maternal and fetal factors found to be significantly different across the groups (maternal obesity, AMA, FGR). Given the smaller sample size of patients with typical PPHN, a single variable for vascular diseases of pregnancy (inclusive of gestational diabetes, gestational hypertension, and/or pre-eclampsia) was included in the multivariable model. Results were reported as an odds ratio with a 95% confidence interval. Statistical significance was set at a p-value <0.05. All analyses were performed using Stata SE (Statacorp 2021. Stata Statistical Software: Release 17).

## 3. Results

A total of 106 placentas were included in the analysis: 53 cases with PPHN, 22.6% (n = 12) with typical PPHN and 77.4% (n = 41) with developmental PPHN, and 53 controls. Maternal gravidity, infant gestational age and infant sex were similar across groups. More mothers in the PPHN group compared to controls had gestational hypertension (32.1% vs 15.1%, p = 0.04), obesity (37.7% vs 12.0%, p < 0.01), and fetal growth restriction (20.8% vs 6.0%, p = 0.03). Infants with PPHN had higher rates of neonatal intensive care unit (NICU) admission (100% vs 22.6%, p < 0.01) and increased mortality (17.0% vs 0%, p < 0.01). (Table 2).

Placental lesions occurred more frequently in PPHN placentas than in controls (92.4% vs 60.4%, p < 0.01). Fig. 1 shows the placental pathology associated with PPHN, including lesions of fetal vascular malperfusion, placental inflammation (both maternal and fetal inflammatory response), meconium, and chorangiosis. In multivariable logistic regression modeling, all associations remained significant when adjusting for potential confounding maternal and fetal factors (Table 3). The most common lesion of fetal vascular malperfusion was avascular villi (22.6% of PPHN placentas versus 5.7% of control placentas, p = 0.01). Further, chorangiosis, a finding of increased capillary growth in the terminal chorionic villi secondary to hypoxia, was only found in PPHN placentas (7.6%, p = 0.04).

Signs of placental inflammation were common in the PPHN group, specifically 52.8% of PPHN placentas had evidence of a maternal inflammatory response (p = 0.03) and 43.4% had evidence of a fetal inflammatory response (p < 0.01). Overall, PPHN placentas had an adjusted OR of 4.43 (1.71 – 11.46) for placental inflammation compared to control placentas. Additionally, meconium was documented in 43.4% of PPHN placentas and 15.1% of controls (p < 0.01); the pattern was suggestive of subacute or chronic meconium exposure as it was seen in the chorion both in the extracellular compartment or inside macrophages.

Stratified exploratory secondary analyses of typical and developmental PPHN compared to controls showed some differences in placental associations across the etiologies of PPHN despite their small sample sizes (Table 4). Placental lesions were more frequent in both etiologies of PPHN compared to controls. Only 4.8% of developmental PPHN placentas had no lesions, while no typical PPHN placenta was without lesions; this compared to 39.6% of control placentas with no lesions present. Using multivariate modeling, in developmental PPHN, there were significant increases in the findings of fetal vascular malperfusion (aOR 3.97 (1.03 – 15.29)), placental inflammation (aOR 3.83 (1.40 – 10.46)) and the fetal inflammatory response (aOR 27.23 (3.19 – 232.39)) compared to control placentas. In typical PPHN, there were also significant increases in placental inflammation (aOR 6.05 (1.30 – 28.10)), and both maternal (aOR 8.27 (1.71 – 40.02)) and fetal (aOR 26.69 (4.73 – 150.41)) inflammatory responses. While the fetal inflammatory response had similar effect sizes in both developmental and typical PPHN, the maternal inflammatory response was more significant in typical PPHN placentas. Fetal vascular malperfusion was not significantly increased in typical PPHN placentas after multivariate adjustment, while the presence of meconium was significantly elevated in only typical PPHN placentas (aOR 17.32 (3.40 – 88.15)).

We performed additional analyses to further understand the relationships between the presence of placental meconium, inflammation (maternal and/or fetal inflammatory response), and maternal and/or fetal vascular malperfusion. In univariate logistic regression modeling, histopathologic meconium was associated with placental inflammation in the PPHN group (OR 4.16 (95% CI 1.13, 15.2)), but not in the control group (OR 0.99 (95% CI 0.21, 4.67)), Fig. 2. Amongst placentas with meconium, in the PPHN group (n = 23), 82.6% (19 of 23) also had placental inflammation compared to only 37.5% (3 of 8) in control placentas. Amongst placentas with inflammation, in the PPHN group (n = 35), 54.3% (19 of 35) also had meconium compared to only 15.0% (3 of 20) in control placentas. Meconium presence did not have an association with maternal and/or fetal vascular malperfusion in either PPHN (n = 26, OR 0.67 (95% CI 0.23, 2.01)) or control placentas (n = 13, OR 0.39 (95% CI 0.04, 3.53)). Inflammation did not have an association with maternal and/or fetal vascular malperfusion in PPHN placentas (OR 1.87 (95% CI 0.59, 5.94)), but it did amongst control placentas (OR 3.73 (95% CI 1.01, 13.78)).

## 4. Discussion

This is the first study that analyzes the placenta to better understand the maternal-fetal environment that precedes a diagnosis of persistent pulmonary hypertension of the newborn. Infants with PPHN have more pathological placental lesions of malperfusion, inflammation, chorangiosis, and meconium than unaffected controls. These findings suggest that hypoxia secondary to malperfusion and inflammation precede PPHN and may be contributory mechanisms to the abnormal pulmonary vascular structure and function seen in PPHN.

Prior studies have used the placenta to investigate the impact of the intrauterine environment on other neonatal pulmonary vascular diseases, such as BPD-associated pulmonary hypertension (BPD-PH). This work has shown that the presence of maternal vascular malperfusion in placentas from extremely premature infants was associated with later development of BPD and, further, findings consistent with severe underperfusion, including decreased villous vascularity, were associated with BPD-PH [5,10]. These data suggest a mechanistic role for intrauterine hypoxia-ischemia in impairing normal lung and vascular development [5]. Our findings in PPHN placentas similarly suggest a higher prevalence of chronic prenatal hypoxia-ischemia, supported by the significant increase in fetal vascular malperfusion lesions and chorangiosis. In addition, findings of inflammation and meconium remained significant even after adjusting for maternal and pregnancy characteristics that can be associated with these findings [1,11–16]. Together, this pathology aligns well with known postnatal mechanisms of pulmonary vascular disease and suggests that intrauterine hypoxia and inflammation could contribute to the abnormal development and function of the pulmonary vasculature in PPHN infants.

The relationship of fetal vascular malperfusion and lung development remains an area of investigation. First, it is possible that severe fetal hypoxia due to placental dysfunction could induce pulmonary vasoconstriction and cause pulmonary vascular remodeling in-utero [8]. It could also alter gene expression in pulmonary endothelial and smooth muscle cells involved in vascular growth and function, such as VEGF, 5-HT, and HIF-1 signaling [17]. Lambs born following chronic intrauterine hypoxia secondary to high-altitude gestation exhibit neonatal pulmonary hypertension, with increased vascular reactivity and important molecular changes, suggesting a detrimental effect of severe fetal hypoxia on the pulmonary vasculature [18]. In our study, the association of fetal vascular malperfusion lesions and PPHN did not reach statistical significance in typical PPHN placentas, yet prior data suggests that a subset of these fetuses are similarly exposed to chronic intrauterine hypoxia and have a more severe neonatal presentation. Thureen and colleagues examined 6 placentas of infants with fatal meconium aspiration syndrome. At autopsy, all 8 newborns had histologic pulmonary vascular remodeling, while 3 of the 6 placentas available had ischemic lesions or frank vessel thrombosis documented [4]. Our findings of increased fetal vascular malperfusion in the developmental PPHN placentas further raises the question as to whether fetuses with developmental conditions leading to PPHN might be more severely affected when their placentas have also shown these changes. Together, these data and our results show an important association that could suggest how severe hypoxia might drive cellular and molecular changes in the developing pulmonary vasculature and could serve as a potential mechanism for vascular remodeling and altered pulmonary vascular reactivity in PPHN. Further investigation is needed to support this mechanistic hypothesis.

Inflammation was also prominent in placentas from infants with PPHN, with the fetal inflammatory response demonstrating a strong association in adjusted analyses for both the developmental and typical PPHN placentas. Inflammation and the presence of circulating immune factors are known to be important in the pathophysiology of pulmonary hypertension by causing pulmonary vascular damage, remodeling, and dysfunction [19–21]. Aggregation of immune cells, especially macrophages, in pulmonary arteries, worsens vascular remodeling by causing maladaptive fibroproliferative changes [22]. Various cytokines and chemokines are increased in patients with pulmonary hypertension, and have been found to interact with key regulators (e.g. prostacyclin) and pathways (e.g. BMPR2 signaling pathway) of normal pulmonary artery adaptation [23]. The association of placental inflammatory lesions with both of our PPHN groups could suggest that mechanisms of inflammation previously described in pulmonary vascular pathophysiology could also have a detrimental effect during pulmonary vascular development when present in the intrauterine environment.

We also found the presence of meconium in the placenta to be significantly associated with PPHN, a relationship that persisted in the typical PPHN placentas after adjustment for factors associated with vascular diseases of pregnancy. In our study, meconium was seen both free-floating and phagocytosed by the chorionic macrophages, and with associated columnar epithelial changes (cellular changes representative of a longer meconium exposure), indicating chronic placental exposure to meconium from the amniotic fluid [24]. Fetal meconium passage with aspiration in the setting of fetal distress is a well-established clinical cause of PPHN, and may further be accompanied by neonatal encephalopathy treated with therapeutic hypothermia, a scenario in which PPHN is prevalent [25]. We found that placentas with meconium were more likely to also exhibit inflammation, however, this relationship was only present in PPHN placentas and not seen in controls. It is difficult to know from our study design whether this association also represents a causal relationship; we could hypothesize that either chronic meconium exposure is a fetal response to inflammation, or conversely, it is acting as a toxin causing inflammation, and is therefore another possible contributing mechanism to PPHN. Among the 6 placentas Thureen and colleagues examined, only 2 had meconium present; both of these placentas also had evidence of malperfusion and one had inflammation [4]. We did not find a relationship between maternal and/or fetal vascular malperfusion and meconium in our PPHN placentas, but there was a significant relationship among the control placentas. These data suggest complex interrelationships between inflammation, meconium, and any type of vascular malperfusion, which could highlight their variable effects on pulmonary vascular remodeling and reactivity depending on other maternal or fetal risk factors for PPHN (Fig. 2).

Overall, this study has multiple strengths. We performed standardized placental pathology review for the purposes of research by a pathologist blinded to the diagnosis of PPHN to reduce observer bias. We minimized selection bias by having equal case and control groups and randomly selecting controls from a baseline population with similar risk factors. Notably, our distribution of PPHN groups is not representative of the general population. As a referral center for fetal anomalies, we had a relatively large number of developmental PPHN cases included in our study, which is not the usual majority etiology of PPHN cases seen in the general public [1,26]. A weakness, as noted, was the limited number of typical PPHN cases which reduced generalizability of this study to non-academic NICUs and made it difficult to draw clinically relevant conclusions as evidenced by our large confidence intervals. Further, our sample sizes, especially for the secondary analyses, are not large enough to detect differences across the subgroups without Type 1 errors; these analyses are mainly exploratory to generate hypotheses for future work. Additionally, typical PPHN cases may not have had a clear indication for requesting a placental pathologic analysis, and so there may be selection bias in this group. Given the single-center and retrospective nature of our study, future multi-center and prospective studies with larger sample sizes are needed to validate our findings.

In conclusion, placentas from infants with PPHN are more likely to have histologic lesions, including fetal vascular malperfusion, inflammation, meconium, and chorangiosis. These placental lesions illustrate that an abnormal maternal-fetal environment marked by hypoxia and inflammation are associated with PPHN, and may be active in both developmental and typical causes of PPHN. Our data suggest that further investigation of the maternal-fetal milieu and its relationship to the clinical presentation of PPHN could provide insight into the mechanisms affecting pulmonary vascular maldevelopment and maladaptation remanifesting as PPHN. Further work is needed to better understand the effect of intrauterine insults and placental findings as it relates to neonatal pulmonary vascular disease.

## Figures and Tables

**Fig. 1.**
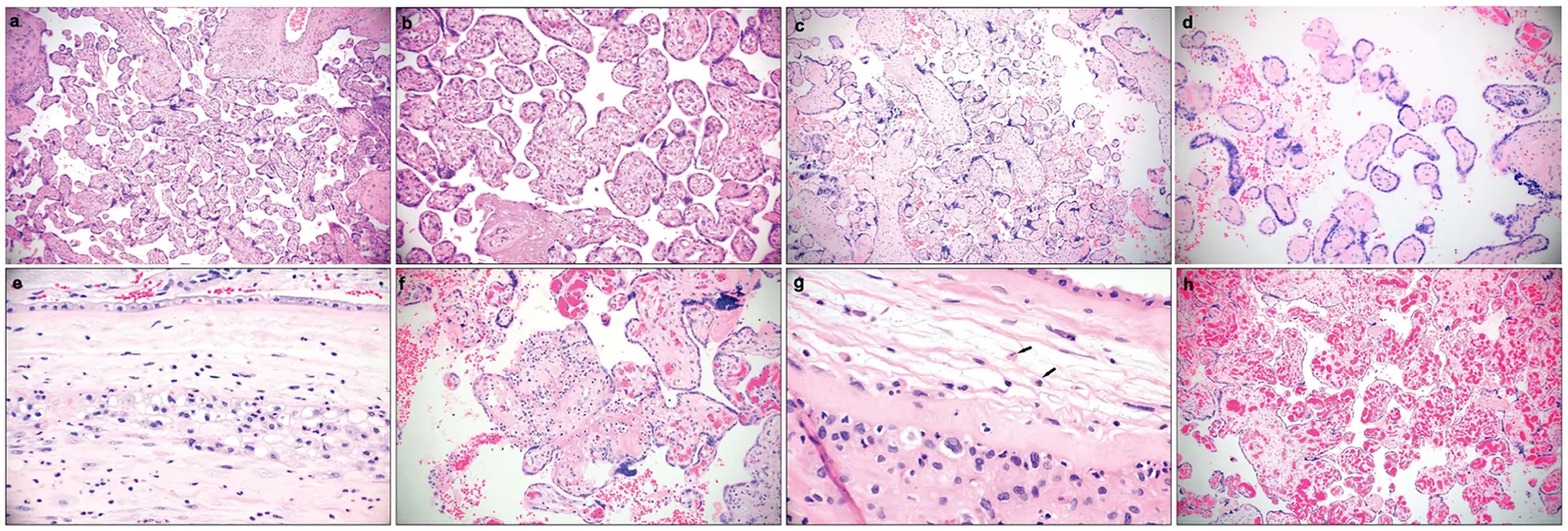
Histologic findings in patients with PPHN stained with hematoxylin and eosin (H&E). **A** and **B:** Control placentas showing chorionic villi with no significant pathologic abnormality (H&E 100x and 200x). **C** and **D:** Findings consistent with fetal vascular malperfusion (FVM) include large focus of contiguous avascular villi with pale fibrotic cores (H&E 100x and 200x). **E:** High-power view of acute chorioamnionitis showing confluent neutrophils within membranes (H&E 400x). **F:** Villitis of unknown etiology (VUE) showing small lymphocytes, monocytes and macrophages in villous stroma (H&E 200x). **G:** Fetal membranes with macrophages (indicated by black arrows) displaying meconium pigment (H&E 600x). **H:** Chorangiosis showing increases capillaries within chorionic villi (H&E 200x).

**Fig. 2.**
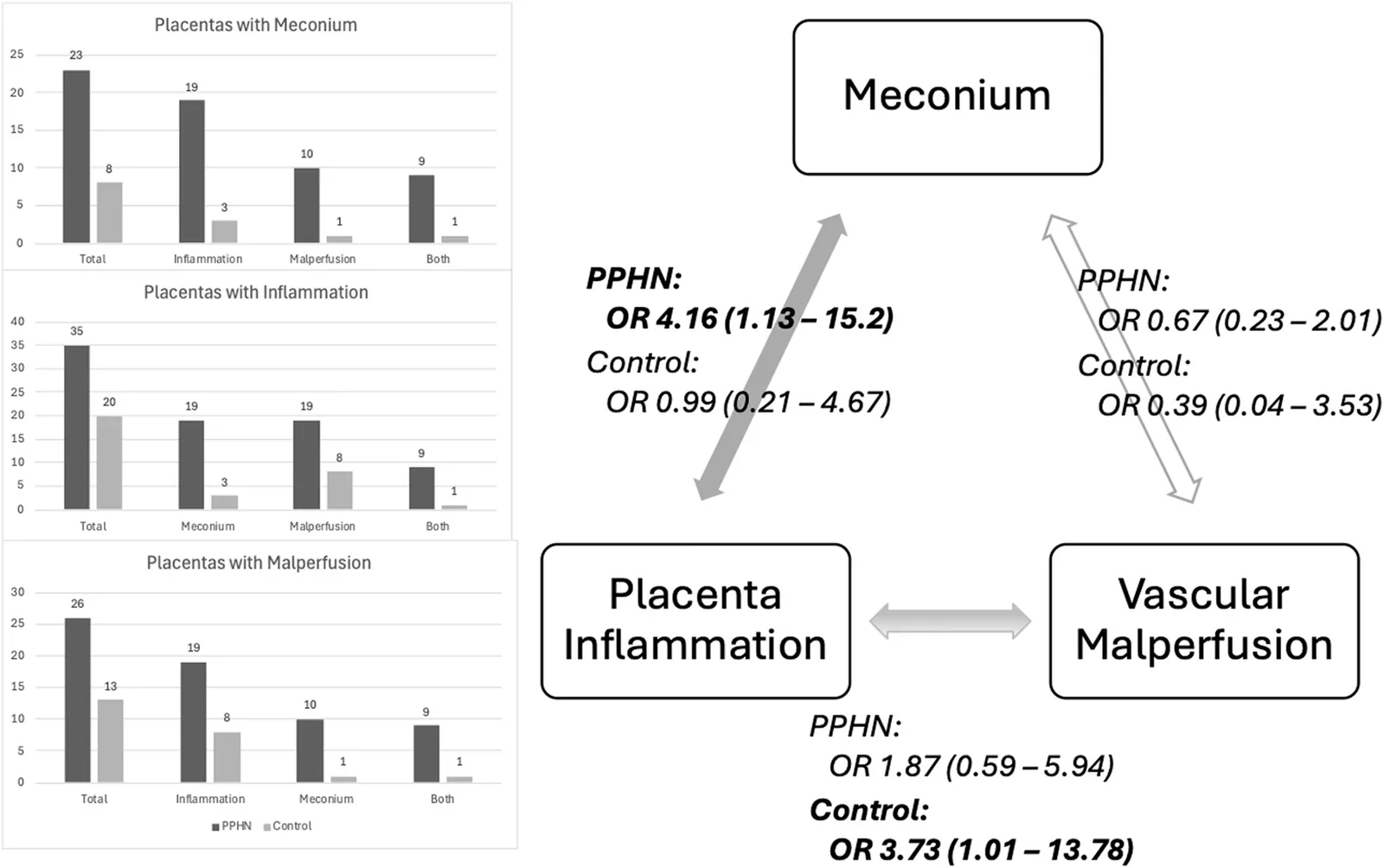
Interrelationships of placental histopathology lesions, stratified by PPHN and control placentas. A. Bar graphs showing counts of placental inflammation and malperfusion among placentas with meconium (top), inflammation (middle) and malperfusion (bottom). B. Proposed relationships between the three significant placental histopathology lesions (meconium, inflammation and malperfusion). Arrows labeled with unadjusted odds ratio and 95% confidence interval calculated for the association between the two placental lesions the arrow connects.

**Table 1:** Description of placental lesions based on the Amsterdam Criteria.

Category	Example of diagnoses
Fetal vascular malperfusion	Vascular thrombi
	Avascular villi
	Villous stromal-vascular karyorrhexis
	Intramural fibrin deposition
	Stem villous obliteration/fibromuscular sclerosis
	Vascular ectasia
Maternal vascular malperfusion	Placental hypoplasia (weight <10th percentile)
	Placental infarct
	Accelerated villous maturation
	Retroplacental hemorrhage
	Distal villous hypoplasia
	Decidual arteriopathy
	Increased syncytial knots
Placental Inflammation	Chronic Deciduitis
	Maternal inflammatory response Acute subchorionitis Acute chorioamnionitis Chorionic plate inflammation
	Fetal inflammatory response Chronic villitis Umbilical phlebitis^a^ Umbilical arteritis^a^ Funisitis^a^
Other placental finding	Meconium
	Chorangiosis
	Intervillous thrombi
	Hyper/Hypocoiled cord^a^

a Only available in cases, controls did not include the umbilical cord.

**Table 2:** Demographic and clinical characteristics of the patient cohort. Comparison of cases versus controls and by etiology of PPHN (developmental and typical).

Characteristic	PPHN (n = 53)	Control	P-value	Developmental PPHN	Typical PPHN	P-value
		(n = 53)		(n = 41)	(n = 12)	
**Maternal age (y)**	**31.2** ± **5.8**	**33.9** ± **3.6**	**< 0.01**	**30.1** ± **5.5**	**34.7** ± **5.6**	**0.01**
Gravidity	2.2 ± 1.4	2.1 ± 1.5	0.56	2.2 ± 1.3	2.2 ± 1.7	0.87
Race						
White	17 (34.0%)	25 (47.2%)		**12 (31.6%)**	**5 (41.7%)**	
Black	2 (4.0%)	2 (3.8%)		**1 (2.6%)**	**1 (8.3%)**	
Asian	8 (16.0%)	8 (15.1%)		**6 (15.8%)**	**2 (16.7%)**	
Multiple/Other	23 (46.0%)	18 (34.9%)	0.07	**19 (50.0%)**	**4 (33.3%)**	**0.03**
Maternal Comorbidities						
Chronic Hypertension	3 (5.7%)	3 (5.7%)	1.00	2 (4.9%)	1 (8.3%)	0.65
**Gestational Hypertension**	**17 (32.1%)**	**9 (15.1%)**	**0.04**	**9 (22.0%)**	**8 (66.7%)**	**< 0.01**
Preeclampsia	7 (13.2%)	2 (4.0%)	0.10	4 (9.8%)	3 (25.0%)	0.17
Pregestational Diabetes	2 (3.8%)	1 (1.9%)	0.56	2 (4.9%)	0	0.44
Gestational Diabetes	2 (3.8%)	5 (9.4%)	0.24	2 (4.9%)	0	0.44
**BMI > 30 kg/m2**	**20 (37.7%)**	**6 (12.0%)**	**< 0.01**	17 (41.5%)	3 (25.0%)	0.30
Substance Use	2 (3.8%)	0	0.17	1 (2.4%)	1 (8.3%)	0.35
Smoking	4 (7.6%)	0	0.05	3 (7.3%)	1 (8.3%)	0.91
IVF Pregnancy	3 (5.7%)	8 (16.3%)	0.08	2 (4.9%)	1 (8.3%)	0.65
**Advanced Maternal Age**	**15 (28.3%)**	**26 (49.1%)**	**0.03**	9 (22.0%)	6 (50.0%)	0.06
No Comorbidities	11 (20.8%)	15 (28.3%)	0.37	9 (22.0%)	2 (16.7%)	0.69
Fetal/Pregnancy Conditions						
Multiple Pregnancy	3 (5.7%)	3 (5.7%)	1.00	3 (7.3%)	0	0.34
Oligohydramnios	6 (11.3%)	1 (2.1%)	0.07	5 (12.2%)	1 (8.3%)	0.71
**Fetal Growth Restriction**	**11 (20.8%)**	**3 (6.0%)**	**0.03**	**11 (26.8%)**	**0**	**0.04**
Chorioamnionitis	9 (17.0%)	6 (12.0%)	0.47	5 (12.2%)	4 (33.3%)	0.09
Delivery Outcomes						
**Gestational Age**	38.3 ± 1.5	38.7 ± 1.2	0.10	**37.9** ± **1.2**	**39.6** ± **1.8**	**< 0.01**
C-Section Delivery	24 (45.3%)	21 (39.6%)	0.56	16 (39.0%)	8 (66.7%)	0.09
Preterm Delivery (<37 wk)	6 (11.3%)	2 (3.8%)	0.14	5 (12.2%)	1 (8.3%)	0.71
Small for Gestational Age	10 (18.9%)	5 (9.4%)	0.16	8 (19.5%)	2 (16.7%)	0.83
Infant Sex (Male)	30 (56.6%)	29 (54.7%)	0.85	24 (58.5%)	6 (50.0%)	0.60
**APGAR 1-min**	**4.6** ± **2.7**	**7.3** ± **1.8**	**< 0.01**	**5.1** ± **2.5**	**3** ± **3.1**	**0.02**
**APGAR 5-min**	**5.7** ± **2.5**	**8.6** ± **1.0**	**< 0.01**	5.9 ± 2.6	5.3 ± 2.2	0.53
**Cord Blood PH**	7.25 ± 0.10	7.05 ± 1.19	0.26	**7.28** ± **0.06**	**7.15** ± **0.12**	**< 0.01**
**Cord Blood BE**	−3.5 ± 4.3	−4.3 ± 3.2	0.34	−**2.1** ± **3.4**	−**7.4** ± **4.5**	**< 0.01**
Neonatal Outcomes^a^						
CHD	21 (39.6%)	0	<0.01	21 (51.2%)	-	-
CDH	23 (43.4%)	0	<0.01	23 (56.1%)	-	-
Fetal Renal Disease	3 (5.7%)	0	0.08	3 (7.3%)	-	-
Meconium Aspiration Syndrome	7 (13.2%)	3 (5.7%)	0.46	-	7 (58.3%)	-
Sepsis	1 (1.9%)	0	0.32	0	1 (8.3%)	0.06
Idiopathic PPHN	5 (9.4%)	0	0.02	-	5 (41.7%)	-
ECMO	7 (13.2%)	0	<0.01	5 (12.2%)	2 (16.7%)	0.69
**NICU Admission**	**53 (100%)**	**12 (22.6%)**	**< 0.01**	41 (100%)	12 (100%)	-
**Death**	**9 (17.0%)**	**0**	**< 0.01**	7 (17.1%)	2 (16.7%)	0.97

Abbreviations: BMI: body mass index, IVF: in-vitro fertilization, APGAR: appearance, pulse, grimace, activity, respiration, BE: base excess, CHD: congenital heart disease, CDH: congenital diaphragmatic hernia, ECMO: extracorporeal membranous oxygenation, NICU: neonatal intensive care unit.

a Some infants had multiple diagnoses.

**Table 3:** Univariate and multivariate analyses of placental histopathology for cases versus controls. Univariate analyses by chi-squared and univariate logistic regression. Multivariate analyses adjusted for gestational hypertension, maternal obesity, advanced maternal age, and fetal growth restriction.

	PPHN (n = 53)	Control (n = 53)	P-value	Unadjusted OR	Adjusted OR
Fetal Vascular Malperfusion	16 (30.2%)	5 (9.4%)	<0.01	4.15 (1.39 – 12.37)	3.79 (1.07 – 13.49)
Maternal Vascular Malperfusion	18 (34.0%)	9 (17.0%)	0.05	2.51 (1.01 – 6.28)	2.28 (0.82 – 6.38)
**Placental Inflammation**	**35 (66.0%)**	**20 (37.7%)**	**< 0.01**	**3.20 (1.45–7.10)**	**4.43 (1.71–11.46)**
**Maternal Inflammatory Response**	**28 (52.8%)**	**17 (32.1%)**	**0.03**	**2.37 (1.08–5.22)**	**2.79 (1.13–6.90)**
**Fetal Inflammatory Response**	**23 (43.4%)**	**3 (5.7%)**	**< 0.01**	**12.78 (3.53–46.21)**	**41.5 (4.97–346.79)**
**Other Findings**					
**Meconium**	**23 (43.4%)**	**8 (15.1%)**	**< 0.01**	**4.31 (1.71–10.90)**	**3.74 (1.35–10.40)**
Chorangiosis	**4 (7.6%)**	**0**	**0.04**	-	-
Intervillous thrombi	4 (7.6%)	2 (3.8%)	0.40	2.08 (0.36 – 11.88)	3.96 (0.58 – 26.95)
**No Lesions**	**4 (7.6%)**	**21 (39.6%)**	**< 0.01**	**0.12 (0.04–0.40)**	**0.11 (0.03–0.41)**

**Table 4:** Stratified univariate and multivariate analyses of placental histopathology by etiology of PPHN (developmental and typical versus controls). Univariate analyses by chi-squared and univariate logistic regression. Multivariate analyses for developmental PPHN adjusted for maternal obesity, advanced maternal age and fetal growth restriction, and for typical PPHN adjusted for a composite variable of vascular disease of pregnancy (gestational diabetes, gestational hypertension and/or pre-eclampsia).

a.	Developmental PPHN (n = 41)	Control (n = 53)	P-value	Unadjusted OR	Adjusted OR
**Fetal Vascular Malperfusion**	**12 (29.3%)**	**5 (9.4%)**	**0.01**	**3.97 (1.27–12.42)**	**3.97 (1.03–15.29)**
Maternal Vascular Malperfusion	14 (34.2%)	9 (17.0%)	0.06	2.53 (0.97 – 6.65)	1.75 (0.59 – 5.19)
**Placental Inflammation**	**26 (63.4%)**	**20 (37.7%)**	**0.01**	**2.86 (1.23–6.65)**	**3.83 (1.40–10.46)**
Maternal Inflammatory Response	19 (46.3%)	17 (32.1%)	0.16	1.83 (0.79 – 4.25)	2.14 (0.82 – 5.60)
**Fetal Inflammatory Response**	**15 (36.6%)**	**3 (5.7%)**	**< 0.01**	**9.62 (2.55–36.25)**	**27.23 (3.19–232.39)**
**Other Findings**	**14 (34.2%)**	**8 (15.1%)**	**0.03**	**2.92 (1.08–7.86)**	2.51 (0.84 – 7.49)
	2 (4.9%)	0	0.10	-	-
Meconium -Chorangiosis -Intervillous thrombi	3 (7.3%)	2 (3.8%)	0.45	2.01 (0.32 – 12.65)	3.53 (0.48 – 26.01)
**No Lesions**	**4 (9.8%)**	**21 (39.6%)**	**< 0.01**	**0.16 (0.05–0.53)**	**0.17 (0.05–0.62)**
b.	Typical PPHN (n = 12)	Control (n = 53)	P-value	Unadjusted OR	Adjusted OR
**Fetal Vascular Malperfusion**	**4 (33.3%)**	**5 (9.4%)**	**0.03**	**4.80 (1.06–21.79)**	4.78 (0.92 – 24.81)
**Maternal Vascular Malperfusion**	4 (33.3%)	9 (17.0%)	0.20	2.44 (0.60 – 9.89)	4.05 (0.79 – 20.68)
**Placental Inflammation**	**9 (75.0%)**	**20 (37.7%)**	**0.02**	**4.95 (1.20–20.47)**	**6.05 (1.30–28.10)**
**Maternal Inflammatory Response**	**9 (75.0%)**	**17 (32.1%)**	**< 0.01**	**6.35 (1.52–26.5)**	**8.27 (1.71–40.02)**
**Fetal Inflammatory Response**	**8 (66.7%)**	**3 (5.7%)**	**< 0.01**	**33.33 (6.26–177.55)**	**26.69 (4.73–150.41)**
**Other Findings**	**9 (75.0%)**	**8 (15.1%)**	**< 0.01**	**16.88 (3.74–76.21)**	**17.32 (3.40–88.15)**
	**2 (16.7%)**	**0**	**< 0.01**	-	-
**Meconium** Chorangiosis Intervillous thrombi	1 (8.3)	2 (3.8%)	0.50	2.32 (0.19 – 27.89)	6.17 (0.43 – 89.34)
**No Lesions**	**0**	**21 (39.6%)**	**< 0.01**	-	-

## ABBREVIATIONS AND ACRONYMS

AMA: advanced maternal age
BPD: bronchopulmonary dysplasia
BPD-PH: bronchopulmonary dysplasia associated pulmonary hypertension
CDH: congenital diaphragmatic hernia
FGR: fetal growth restriction
NICU: neonatal intensive care unit
PPHN: persistent pulmonary hypertension of the newborn
SGA: small for gestational age
